# Resting low-frequency EEG correlates of adult ADHD trait symptoms beyond internalizing symptoms in young adults

**DOI:** 10.3389/fnhum.2026.1878112

**Published:** 2026-07-29

**Authors:** Hongqin Du, Xiaomeng Gao, Yining Kou, Huili Xing, Ronglian Zheng, Yihan Wu, Bo Zhang, Xiang Li, Meng Zhang

**Affiliations:** 1 Department of Psychology, Henan Medical University, Xinxiang, Henan, China; 2 College of Education, Shanghai Normal University, Shanghai, China; 3 Jing Hengyi School of Education, Hangzhou Normal University, Hangzhou, China; 4 Sichuan Academy of Medical Sciences and Sichuan Provincial People's Hospital, Chengdu, Sichuan, China; 5 Henan Provincial People's Hospital, Zhengzhou, Henan, China

**Keywords:** adult ADHD traits, delta power, internalizing symptoms, low-frequency EEG, posterior-occipital EEG, resting-state EEG, theta/beta ratio

## Abstract

**Introduction:**

Intrinsic EEG oscillations provide a non-invasive window into arousal- and attention-related individual differences, but resting low-frequency EEG correlates of adult attention-deficit/hyperactivity disorder (ADHD) trait symptoms remain incompletely characterized in non-clinical young adults.

**Methods:**

We analyzed eyes-closed resting-state EEG from a cohort of 337 Chinese undergraduates with usable EEG data; the primary analysis included 334 participants. ADHD trait symptoms were assessed dimensionally with a 26-item adult ADHD self-report scale. The prespecified EEG family comprised log posterior-occipital delta power, log posterior delta power, log global delta power, and frontocentral log theta/beta ratio. The primary HC3 regression adjusted for age, sex, trait anxiety, depressive symptoms, and Beck Anxiety Inventory total score, with false-discovery-rate correction across the planned EEG family.

**Results:**

Higher ADHD trait symptoms were associated with lower posterior-occipital delta power [β = 0.114, 95% CI (0.199, 0.029), *p* = 0.008]. Posterior delta, global delta, and frontocentral theta/beta ratio showed convergent negative associations after false-discovery-rate correction. The posterior-occipital delta association remained negative and statistically significant across extended covariate models and robustness analyses. Across 64 reasonable covariate specifications, all coefficients were negative and 65.6% reached *p* < 0.05.

**Discussion:**

Adult ADHD trait symptoms were modestly associated with lower posterior-occipital and broader low-frequency resting EEG power after accounting for internalizing symptoms. The findings support a stable group-level dimensional association within this sample, but not individual-level screening, diagnostic support, risk classification, or causal inference.

## 1 Introduction

Attention-deficit/hyperactivity disorder (ADHD) is a neurodevelopmental condition characterized by persistent inattention, hyperactivity, and impulsivity (American Psychiatric Association, 2013; Faraone et al., 2021). Although ADHD is often identified in childhood, symptoms frequently persist into adolescence and adulthood and are associated with difficulties in attention regulation, executive control, academic functioning, and emotional adjustment (Biederman et al., 2011; Kessler et al., 2006; Kooij et al., 2019; Sibley et al., 2017). In non-clinical young adults, ADHD-related traits can be studied dimensionally, capturing individual differences in attentional instability, restlessness, impulsivity, emotional lability, and self-concept difficulties without implying clinical diagnosis (Kessler et al., 2005; Song et al., 2021).

A dimensional resting-EEG approach is useful for this problem because ADHD-related traits vary continuously in the general population and involve cognitive domains such as sustained attention, vigilance, inhibitory control, and arousal regulation (Faraone et al., 2021; Lenartowicz et al., 2018). Recent psychometric work in adolescents and young adults also supports dimensional self-report measurement of ADHD-related symptom variation (Vajsz et al., 2024). Rather than asking whether EEG can classify ADHD, the present study asked whether low-frequency resting activity is associated with individual differences in adult ADHD trait symptoms after accounting for internalizing symptoms and other relevant covariates. This distinction is important because trait-level symptoms in a non-clinical sample may reveal subtle oscillatory correlates without implying diagnostic utility (Adamou et al., 2020; Lenartowicz and Loo, 2014; Loo and Makeig, 2012).

A large ADHD EEG literature has focused on theta/beta ratio, traditionally interpreted as reflecting a balance between slower activity and beta-band control-related activity. However, evidence for theta/beta ratio as a diagnostic marker has become increasingly mixed. Individual studies have compared theta/beta ratio and related quantitative EEG measures between ADHD and control groups and examined their behavioral correlates and diagnostic performance (Ogrim et al., 2012; Markovska-Simoska and Pop-Jordanova, 2017). Beyond diagnostic applications, frontal theta/beta ratio has also been examined in relation to attentional control, response inhibition, and mind wandering (Putman et al., 2010; Angelidis et al., 2016; van Son et al., 2019). Related work has demonstrated genetic overlap among evoked frontocentral theta-phase variability, reaction-time variability, and ADHD symptoms (McLoughlin et al., 2014). Meta-analytic and review work indicates substantial heterogeneity across age, ADHD presentation, recording condition, preprocessing pipeline, and clinical status (Arns et al., 2013; Lenartowicz and Loo, 2014; Loo and Makeig, 2012; Saad et al., 2018). A recent systematic review of adult ADHD EEG and ERP studies, which searched the literature through August 2024 and included 68 studies, further underscores both the continued interest in resting spectral power and the absence of a simple adult ADHD EEG marker (Su et al., 2025). Recent young-adult QEEG work likewise continues to implicate frequency-specific power and topographic features while emphasizing the importance of recording context and sample characteristics (Yoon et al., 2024). Adult ADHD studies have therefore been especially cautious: some work indicates that spectral power may carry more useful information than theta/beta ratio alone, whereas other work emphasizes the limited diagnostic specificity of resting EEG summaries (Adamou et al., 2020; Kiiski et al., 2020; Boxum et al., 2025). Thus, adult ADHD-related EEG variation is better approached as a set of modest oscillatory correlates than as a single diagnostic EEG signature.

Low-frequency EEG power is important in this context. Delta and theta activity are related to arousal, vigilance, developmental state, and large-scale cortical organization, but their interpretation depends on age, state, region, and analytic pipeline (Barry et al., 2003; Buzsáki, 2006; Klimesch, 1999; Knyazev, 2012; Newson and Thiagarajan, 2019; Su et al., 2025). In ADHD, the historical emphasis on theta/beta ratio has been tempered by evidence that the ratio is heterogeneous and not sufficiently specific for diagnosis (Arns et al., 2013; Lenartowicz and Loo, 2014; Saad et al., 2018; Boxum et al., 2025). A broader low-frequency family that includes posterior-occipital and broader posterior delta power together with frontocentral theta/beta balance is therefore better suited to a dimensional resting-oscillation analysis.

ADHD traits are also strongly entangled with internalizing symptoms. Trait anxiety, depressive symptoms, and BAI-indexed somatic anxious arousal can overlap with inattention, restlessness, emotional instability, and subjective cognitive difficulties in adult ADHD and related trait presentations (Faraone et al., 2021; Kessler et al., 2006; Kooij et al., 2010; Kooij et al., 2019). Testing EEG correlates of ADHD traits therefore requires asking whether oscillatory associations are detectable beyond this broader internalizing profile, rather than treating ADHD symptom scores as isolated questionnaire variance.

## 2 Materials and methods

### 2.1 Participants and procedure

Participants were undergraduates recruited from several colleges in Xinxiang, China, as part of an ongoing multimodal project on psychological phenotypes and resting-state EEG. Participants completed a self-report battery and an approximately 6-min eyes-closed resting EEG recording in a quiet laboratory setting. After duplicate EEG records and quality-control exclusions were removed, 337 participants had usable resting-state frequency-domain EEG data and constituted the EEG cohort. The mean age was 18.30 years (SD = 0.84), and 95 participants were male (28.2%). Because only biological sex was recorded at enrollment, sex is reported as male/female. The female-predominant sex distribution and narrow undergraduate age band are addressed as generalizability constraints in the Limitations. Written informed consent was obtained before data collection. The study protocol was approved by the institutional ethics committee and complied with the Declaration of Helsinki.

### 2.2 Adult ADHD trait symptoms

Adult ADHD trait symptoms were assessed with a 26-item adult ADHD self-report scale corresponding to the short self-report format of the Conners Adult ADHD Rating Scales (CAARS-S: S; Conners et al., 1999). Items are rated from 0 to 3 and cover inattention/memory problems, hyperactivity/restlessness, impulsivity/emotional lability, and self-concept problems. Higher scores indicate higher adult ADHD trait symptom burden. Previous studies have evaluated the psychometric properties, factor structure, measurement invariance, and diagnostic accuracy of the CAARS and its short forms in adult, emerging-adult, and postsecondary samples (Amador-Campos et al., 2014; Harrison et al., 2019; Wu et al., 2023). A score of 39 or higher was used only as a descriptive risk-threshold indicator, not as a clinical diagnosis. The total score was designated as the primary outcome. Subscale analyses were supportive because some subscale reliabilities were lower than the total-scale reliability.

### 2.3 Psychological covariates and sensitivity variables

Trait anxiety was assessed with the trait form of the State–Trait Anxiety Inventory (STAI-T; Spielberger et al., 1983), depressive symptoms with the Zung Self-Rating Depression Scale (SDS; Zung, 1965), and anxious arousal with the Beck Anxiety Inventory total score (BAI; Beck et al., 1988). Because the BAI total score includes many somatic and panic-related anxiety symptoms, we describe it as an index of somatic anxious arousal; it was not treated as a separate BAI somatic-anxiety subscale. These variables were specified *a priori* as internalizing-symptom covariates. Sensitivity variables included mobile-phone addiction tendency, internet addiction tendency, Raven reasoning, college entrance examination score, Big Five personality traits, general self-efficacy, and positive affect. These variables were used to test whether the EEG association could be explained by digital-addiction tendencies, broad personality, self-efficacy, affective state, or cognitive performance.

### 2.4 EEG acquisition and preprocessing

Resting EEG was recorded from 64 Ag/AgCl scalp electrodes positioned according to the international 10–20 system using a Neuroscan system at 500 Hz, with Cz as the online reference. Electrode impedances were maintained below 5 kOhm. Horizontal and vertical electrooculographic channels were recorded to aid artifact identification. Participants were instructed to remain awake, relax, minimize movement, and keep their eyes closed. The eyes-closed condition was selected because it provides a low-sensory-input resting state that is commonly used to estimate intrinsic posterior low-frequency activity while reducing visual stimulation and eye-movement burden. We recognize that eyes-open and task-based recordings may be more sensitive for some theta/beta or executive-control hypotheses; the present design was therefore optimized for resting low-frequency delta power rather than for task-evoked attentional dynamics.

Preprocessing followed the laboratory’s standardized resting-state workflow in EEGLAB (Delorme and Makeig, 2004). Continuous EEG was band-pass filtered, gross artifacts were removed by visual inspection, and extended Infomax independent component analysis was used to identify and remove ocular, muscular, and other stereotyped non-neural components (Lee et al., 1999). Cleaned data were re-referenced, segmented into consecutive 2-s epochs, and screened using an amplitude criterion with visual confirmation. Participants were retained only if at least 4 min of artifact-free resting data remained. Frequency-domain features were extracted from artifact-free epochs using a complex Morlet wavelet approach (Cohen, 2019).

### 2.5 EEG variables

The prespecified low-frequency EEG family comprised log posterior-occipital delta power, log posterior delta power, log global delta power, and frontocentral log theta/beta ratio. The primary EEG marker was log posterior-occipital delta power, defined as the natural-log-transformed mean delta power across PO3, POz, PO4, O1, Oz, and O2. The broader posterior delta ROI included parietal, parieto-occipital, and occipital electrodes (P7, P5, P3, P1, Pz, P2, P4, P6, P8, PO7, PO5, PO3, POz, PO4, PO6, PO8, O1, Oz, and O2), whereas global delta was averaged across all available scalp electrodes. Frontocentral theta/beta ratio was included as a conventional ADHD-relevant oscillatory balance metric. Broader exploratory specificity analyses examined additional regional and frequency-band summaries. The frontocentral theta/beta ratio was calculated as the natural log of the mean theta power divided by mean beta power across F1, Fz, F2, FC1, FCz, and FC2.

### 2.6 Statistical analysis

The primary regression modeled ADHD total score as a continuous outcome and log posterior-occipital delta power as the focal EEG predictor, adjusting for age, sex, STAI-T trait anxiety, SDS depressive symptoms, and BAI total score. Zero-order associations between continuous variables were summarized with Pearson correlations because the primary question concerned linear associations among continuous trait and EEG measures; partial Spearman residual correlations were retained as rank-based robustness checks. Continuous variables were standardized before regression, whereas sex was modeled as a binary covariate. Linear models used HC3 robust standard errors because HC3 inference reduces sensitivity to heteroscedasticity and influential leverage patterns in observational individual-differences data. Planned low-frequency EEG family analyses applied the Benjamini-Hochberg false-discovery-rate correction (Benjamini and Hochberg, 1995) across the four prespecified EEG predictors: posterior-occipital delta, posterior delta, global delta, and frontocentral theta/beta ratio. Incremental variance explained by the EEG predictor was calculated relative to the covariate-only model within the same sample.

**Figure 1:**
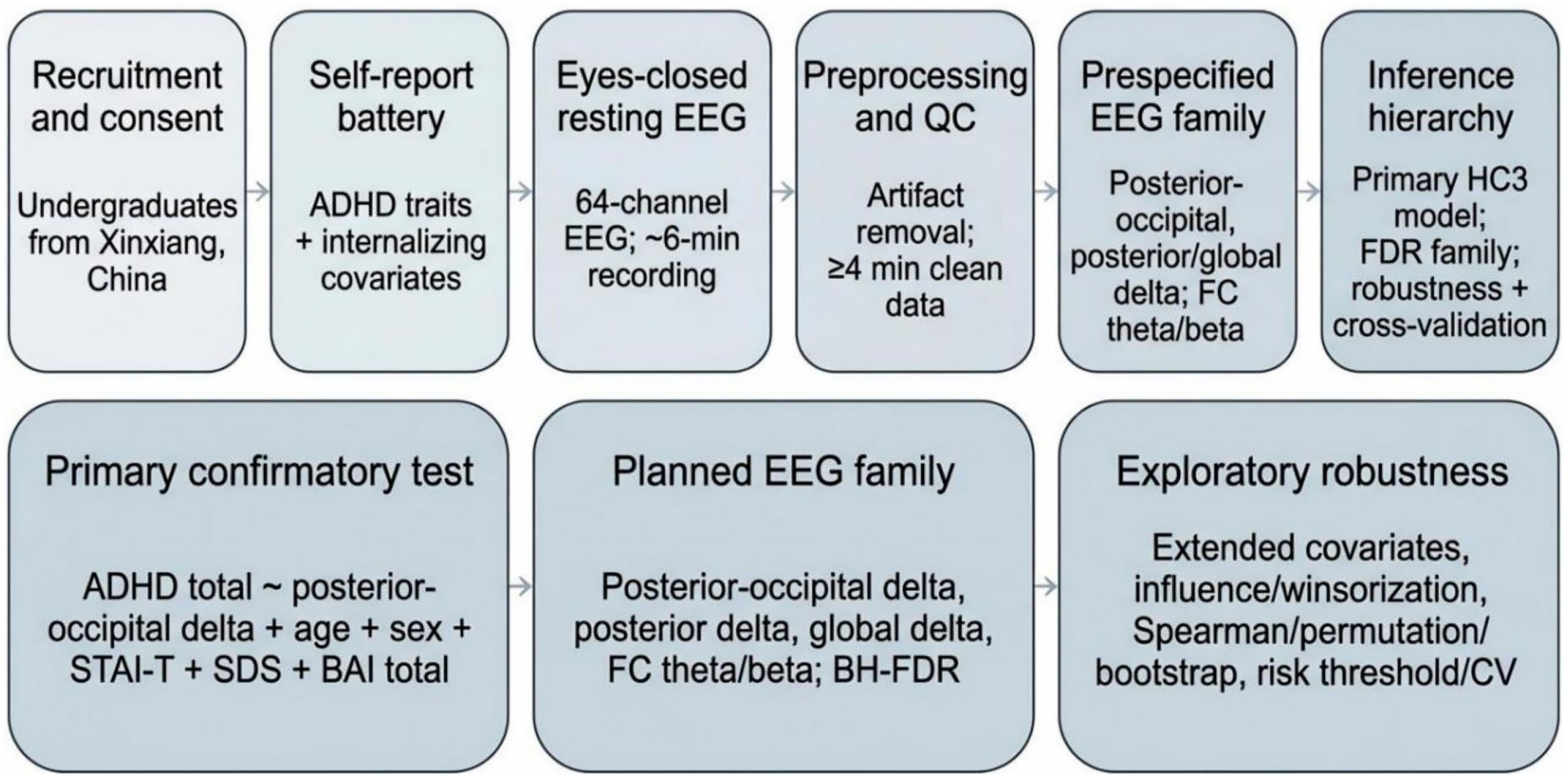
Study framework and analysis pipeline. The schematic distinguishes the primary confirmatory model from the planned low-frequency EEG-family analysis and exploratory robustness/sensitivity analyses.

Sensitivity analyses examined models additionally adjusted for digital-addiction tendencies, cognitive-performance variables, Big Five traits, general self-efficacy, and positive affect. Robustness checks included influence-point exclusion using Cook’s distance, 1%/99% winsorization, partial Spearman correlation, nonparametric permutation testing, bootstrap confidence intervals, sex interaction, and quadratic effects. These analyses were specified to evaluate stability across distributional assumptions, influential observations, nonparametric rank structure, resampling uncertainty, and alternative functional forms rather than to create additional primary endpoints. We also conducted a specification curve across 64 reasonable covariate specifications defined by combinations of internalizing symptoms, digital-addiction tendencies, cognitive-performance variables, Big Five traits, general self-efficacy, and positive/negative affect. Risk-threshold analyses tested the descriptive ADHD > = 39 grouping but were not interpreted as diagnostic classification. All secondary analyses outside the main inferential chain are reported in the Supplementary material, including ADHD subscale models, broad EEG specificity tests, robustness analyses, spatial collinearity checks, outcome-specificity analyses, risk-threshold models, and cross-validation summaries (Supplementary Tables S1–S6 and Supplementary Figures S1–S3). All analyses were interpreted as associations rather than causal effects (see Figure 1).

## 3 Results

### 3.1 Descriptive statistics and reliability

Table 1 summarizes the sample. ADHD total scores were available for 335 participants (M = 22.17, SD = 11.05), and the total scale showed excellent internal consistency (alpha = 0.903). Subscale reliabilities were acceptable to good for inattention/memory (alpha = 0.721), impulsivity/emotional lability (alpha = 0.795), and self-concept problems (alpha = 0.815), and lower for hyperactivity/restlessness (alpha = 0.558). Thirty-two of 335 participants (9.6%) scored at or above the descriptive ADHD risk threshold of 39. This threshold was used only to describe sample distribution and was not interpreted as a clinical diagnosis or individual screening result.

**Table 1:** Descriptive statistics and internal consistency.

Variable	*N*	Mean (SD) or *n* (%)	Cronbach alpha
EEG cohort	337		
Age, years	337	18.30 (0.84)	
Male, n (%)	337	95 (28.2%)	
ADHD total score	335	22.17 (11.05)	0.903
ADHD > = 39, *n* (%)	335	32 (9.6%)	
Inattention/memory	335	5.31 (2.97)	0.721
Hyperactivity/restlessness	335	3.21 (2.14)	0.558
Impulsivity/emotional lability	335	3.47 (2.57)	0.795
Self-concept problems	335	4.77 (3.18)	0.815
Trait anxiety	336	45.61 (7.47)	0.830
Depressive symptoms (SDS)	335	37.44 (7.72)	0.800
BAI total score (somatic anxious arousal)	335	26.83 (6.38)	0.889
Log posterior-occipital delta power	337	0.54 (0.29)	
Log posterior delta power	337	0.61 (0.27)	
Frontocentral log theta/beta ratio	337	0.94 (0.26)	

Values are observed-data summaries. ADHD, attention-deficit/hyperactivity disorder; SDS, Self-Rating Depression Scale; BAI, Beck Anxiety Inventory total score. The ADHD > = 39 risk threshold is descriptive and is not a clinical diagnosis or screening classification.

### 3.2 Primary confirmatory model and planned EEG-family analysis

**Figure 2:**
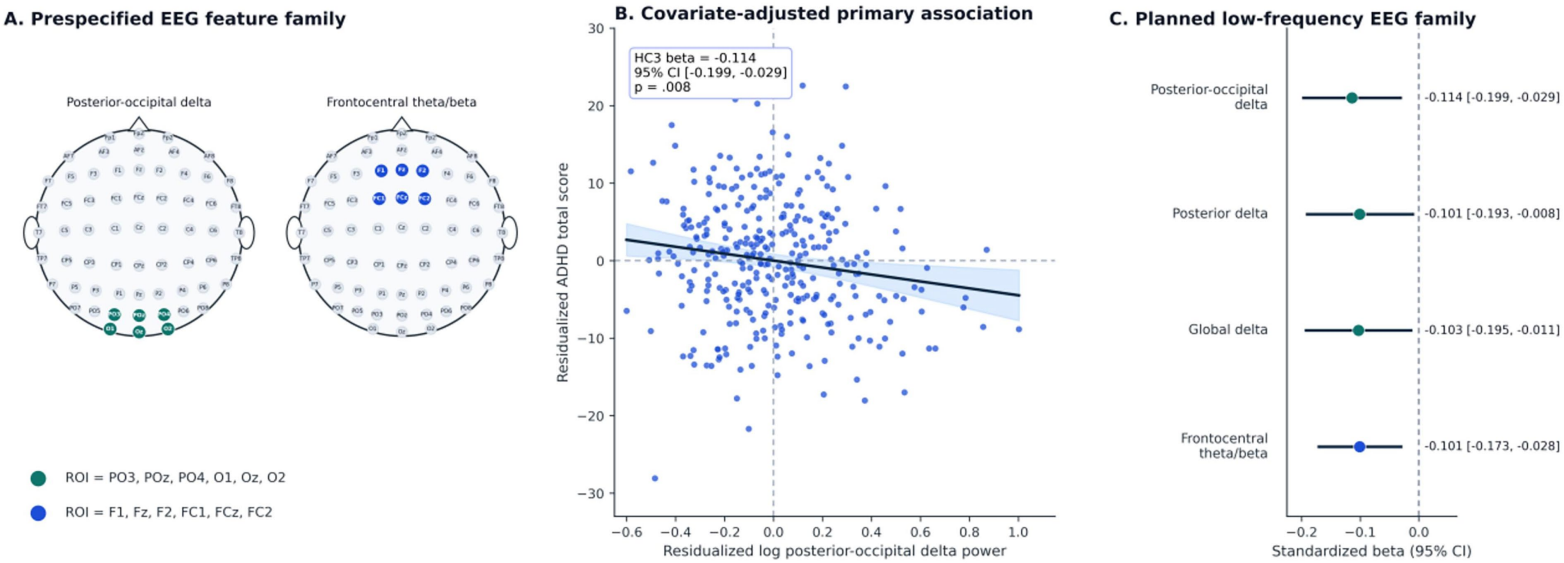
Prespecified low-frequency EEG family and primary association. **(A)** Shows the prespecified posterior-occipital delta ROI and the frontocentral theta/beta electrode set used to define the planned low-frequency EEG family. The posterior-occipital delta ROI comprised PO3, POz, PO4, O1, Oz, and O2; the frontocentral theta/beta feature was defined from F1, Fz, F2, FC1, FCz, and FC2. **(B)** Shows the covariate-adjusted partial association between log posterior-occipital delta power and adult ADHD trait symptoms after adjustment for age, sex, STAI-T trait anxiety, SDS depressive symptoms, and BAI total score. **(C)** Summarizes standardized beta estimates for the planned low-frequency EEG family. Points represent standardized beta coefficients, and bars represent 95% confidence intervals.

In zero-order analyses, the association between ADHD total score and log posterior-occipital delta power was weak (r = −0.072, *p* = 0.187). The association emerged most clearly after adjustment for internalizing symptoms. In the primary confirmatory model controlling for age, sex, STAI-T trait anxiety, SDS depressive symptoms, and BAI total score, higher ADHD total scores were associated with lower log posterior-occipital delta power [beta = −0.114, 95% CI (−0.199, −0.029), *p* = 0.008; Table 2; Figures 2A,B]. Adding log posterior-occipital delta power to the internalizing-symptom model explained an additional 1.2% of variance.

**Table 2:** Planned low-frequency EEG family predicting adult ADHD trait symptoms.

Predictor	*N*	Beta	95% CI	*p*	q (FDR)	ΔR^2^
Posterior-occipital delta	334	−0.114	−0.199 to −0.029	0.008	0.017	0.012
Posterior delta	334	−0.101	−0.193 to −0.008	0.034	0.034	0.009
Global delta	334	−0.103	−0.195 to −0.011	0.029	0.034	0.010
FC theta/beta	334	−0.101	−0.173 to −0.028	0.007	0.017	0.010

All models adjusted for age, sex, STAI-T trait anxiety, SDS depressive symptoms, and BAI total score. Coefficients are standardized betas from HC3 robust regressions. Delta R^2^ denotes incremental variance explained by adding the EEG predictor to the covariate model.

The planned low-frequency EEG family showed convergent negative associations (Table 2; Figure 2C). In addition to posterior-occipital delta power, ADHD total scores were associated with lower posterior delta power, lower global delta power, and lower frontocentral theta/beta ratio after FDR correction within the planned family. These results support a low-frequency oscillatory pattern rather than an isolated single-electrode or single-index finding.

### 3.3 Exploratory extended covariate models and robustness analyses

**Figure 3:**
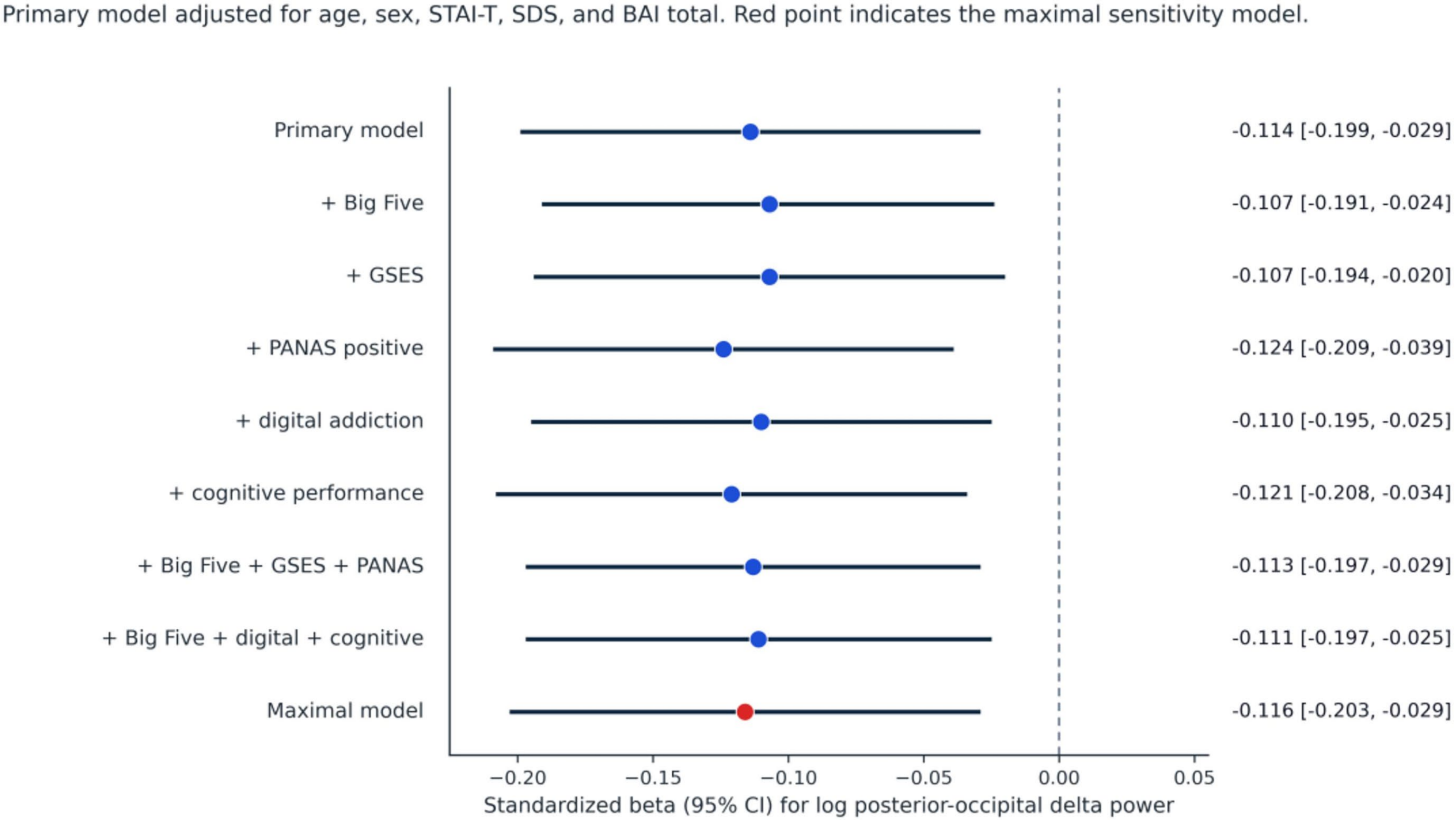
Extended covariate models for the primary posterior-occipital delta association. Points show standardized beta coefficients for log posterior-occipital delta power across the primary and extended covariate models; bars represent 95% confidence intervals. The primary model adjusted for age, sex, STAI-T trait anxiety, SDS depressive symptoms, and BAI total score. Extended rows add the indicated covariate block(s). The red point marks the maximal sensitivity model including the available major psychological, digital-behavioral, and cognitive covariate blocks.

Extended covariate models are summarized in Table 3 and Figure 3. The posterior-occipital delta association remained significant after additional adjustment for digital-addiction tendencies, cognitive-performance variables, Big Five traits, general self-efficacy, positive affect, and combined covariate blocks. In the maximal sensitivity model including the major psychological, digital-behavioral, and cognitive covariate blocks, the association remained negative and significant. These models indicate that the effect was not explained by digital-media tendencies, broad personality, self-efficacy, affective state, or cognitive performance, and they are interpreted as robustness/sensitivity analyses rather than as additional confirmatory endpoints.

**Table 3:** Extended covariate models for posterior-occipital delta power.

Model	*N*	Beta	95% CI	*p*	ΔR^2^
Primary model	334	−0.114	−0.199 to −0.029	0.008	0.012
+ Big five	333	−0.107	−0.191 to −0.024	0.012	0.010
+ GSES	333	−0.107	−0.194 to −0.020	0.016	0.010
+ PANAS positive	334	−0.124	−0.209 to −0.039	0.004	0.014
+ Digital addiction	334	−0.110	−0.195 to −0.025	0.011	0.011
+ Cognitive performance	316	−0.121	−0.208 to −0.034	0.006	0.013
+ Big Five + GSES + PANAS	332	−0.113	−0.197 to −0.029	0.009	0.011
+ Big Five + digital + cognitive	315	−0.111	−0.197 to −0.025	0.012	0.011
Maximal model	314	−0.116	−0.203 to −0.029	0.009	0.012

Each row adds the indicated covariate block to the primary internalizing-symptom model, except the maximal sensitivity model, which includes the available major psychological, digital-behavioral, and cognitive covariate blocks. Coefficients are standardized betas for log posterior-occipital delta power.

Robustness checks supported the stability of the primary association (Supplementary Table S3). Excluding influential observations according to Cook’s distance strengthened the estimate, winsorization yielded a similar effect, the partial Spearman and permutation tests remained significant, and bootstrap confidence intervals did not include zero. No significant sex interaction was observed, and the quadratic term did not support a clear nonlinear association.

### 3.4 Exploratory specification curve and secondary analyses

**Figure 4:**
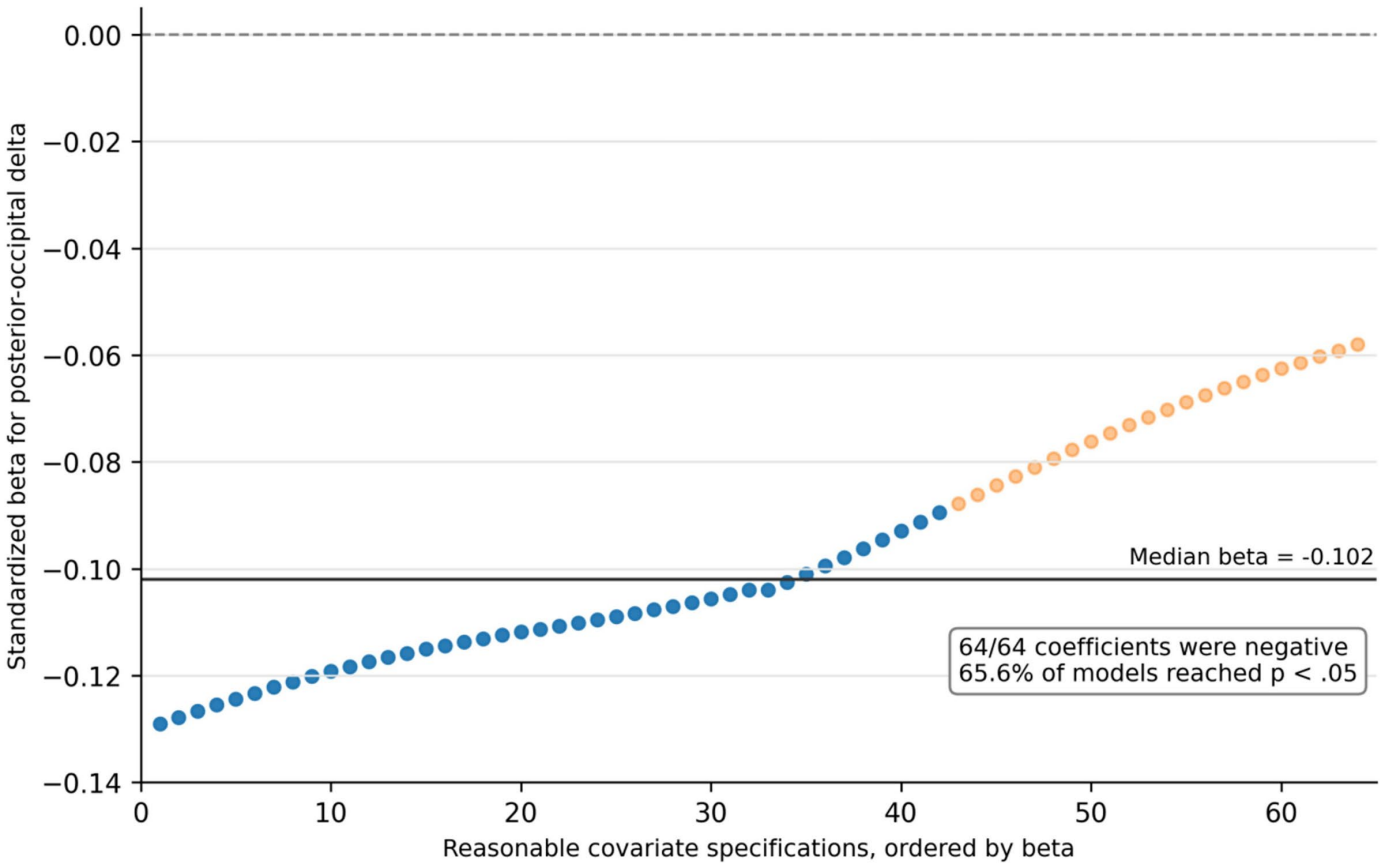
Specification curve across 64 reasonable covariate specifications. Points show standardized beta coefficients for log posterior-occipital delta power ordered by coefficient size. Filled blue points denote specifications with *p* < 0.05, and open orange points denote specifications with *p* > = 0.05. All coefficients were negative, and 65.6% of models reached *p* < 0.05. The horizontal solid line marks the median coefficient and the dashed line marks zero.

The specification curve showed a stable negative direction across analytic choices (Figure 4). Across 64 reasonable covariate specifications, all beta coefficients were negative and 65.6% reached *p* < 0.05. ADHD subscale analyses showed negative coefficients across subscales but were treated as exploratory (Supplementary Table S1). The strongest patterns were observed for hyperactivity/restlessness, impulsivity/emotional lability, and self-concept problems, although the lower reliability of the hyperactivity/restlessness subscale limits interpretation.

Broader EEG specificity analyses indicated that the negative association was most apparent for delta and adjacent low-frequency measures, whereas alpha, beta, and gamma measures were not associated with ADHD total scores (Supplementary Table S2 and Supplementary Figure S2). Posterior-occipital, posterior, and global delta power were highly correlated; simultaneous spatial models showed substantial collinearity among delta indices, so the results should be interpreted as reflecting a posterior/global low-frequency oscillatory pattern rather than a narrowly localized effect (Supplementary Table S4). Outcome-specificity checks suggested that posterior-occipital delta power was not a broad correlate of the major psychological covariates examined (Supplementary Table S5 and Supplementary Figure S3). The ADHD > = 39 risk-threshold logistic model was not significant, and cross-validation showed only a small and imprecise predictive increment (Supplementary Table S6 and Supplementary Figure S1). Thus, the data support a modest dimensional EEG association but not individual-level screening, diagnostic support, or risk classification.

## 4 Discussion

This study tested whether adult ADHD trait symptoms in young adults were associated with resting low-frequency EEG oscillatory activity beyond internalizing symptoms. The main finding was that higher ADHD total scores were modestly associated with lower posterior-occipital and broader posterior delta power after adjustment for trait anxiety, depressive symptoms, and BAI total score indexing somatic anxious arousal. The planned oscillatory-feature family showed convergent negative associations, and the primary posterior-occipital delta result remained directionally stable across extended covariate models, robustness checks, and specification-curve analyses.

These findings situate adult ADHD trait symptoms within the literature on intrinsic low-frequency EEG organization and cognitive vulnerability (Barry et al., 2003; Lenartowicz et al., 2018; Newson and Thiagarajan, 2019; Su et al., 2025). ADHD-related traits involve attention, vigilance, and self-regulatory processes that plausibly depend on the background organization of resting cortical activity (Faraone et al., 2021; Kooij et al., 2019). The present findings do not identify a diagnostic signature, but they suggest that the internalizing-adjusted component of adult ADHD trait symptoms is associated with lower posterior-occipital and broader posterior low-frequency power.

The findings also contribute to the debate around theta/beta ratio and resting EEG in ADHD. The historical emphasis on theta/beta ratio has been tempered by evidence that this metric is heterogeneous and has limited diagnostic utility (Arns et al., 2013; Clarke et al., 2019; Lenartowicz and Loo, 2014; Saad et al., 2018; Boxum et al., 2025). In the present dimensional sample, frontocentral theta/beta ratio showed a negative association within the planned low-frequency family, but the clearest primary effect was observed for posterior-occipital delta power. This pattern supports treating theta/beta ratio as one component of a broader low-frequency oscillatory profile rather than as a standalone ADHD marker.

A central feature of the result is that the zero-order association between ADHD traits and posterior-occipital delta power was weak, whereas the covariate-adjusted association was clearer. This pattern means that the result should be interpreted as an association with the internalizing-adjusted component of adult ADHD trait symptoms. Given the strong overlap between adult ADHD symptoms and internalizing burden in population and clinical studies, such covariate-sensitive patterns should be interpreted cautiously rather than as simple unadjusted trait correlations (Kessler et al., 2006; Kooij et al., 2019; Simon et al., 2009). It does not imply that posterior-occipital delta power has diagnostic utility at the individual level.

Topographic interpretation also requires caution. Posterior-occipital, posterior, and global delta measures were highly intercorrelated, and simultaneous models showed substantial multicollinearity. Because low-frequency oscillations can reflect broad cortical state, arousal-related, and large-scale network organization rather than narrowly localized generators, the present findings should be interpreted as supporting a posterior/global low-frequency emphasis rather than a strictly occipital-specific effect (Buzsáki, 2006; Knyazev, 2012; Newson and Thiagarajan, 2019).

Several features strengthen the interpretability of this study. The sample is relatively large for a resting EEG individual-differences study; the ADHD total score showed excellent internal consistency; the low-frequency EEG family was specified before inferential testing; and the primary result was supported by convergent planned-family, extended-covariate, robustness, and specification-curve analyses. The analytic framework separated the primary confirmatory test from the planned EEG-family analysis and exploratory robustness analyses, reducing the risk that supportive sensitivity checks are mistaken for independent confirmatory endpoints. The extended models are particularly important because they show that the association was not explained by digital-addiction tendencies, broad personality, self-efficacy, affective state, or reasoning ability. These features make the result more informative than a single exploratory EEG-questionnaire correlation: the association is modest, but it is directionally stable, theoretically aligned with attention-related oscillatory vulnerability, and bounded by a transparent analytic framework.

## 5 Limitations and future directions

These strengths should be considered alongside limitations that define the scope of the claim. First, the study was cross-sectional, so the data cannot establish whether lower posterior low-frequency power is an antecedent, consequence, or correlate of ADHD trait symptoms. Second, the sample consisted of non-clinical Chinese undergraduates with a narrow age range and a female-predominant sex distribution; the findings may not generalize to clinically diagnosed ADHD, older adults, sex-balanced samples, or more diverse populations.

The restricted age range is especially relevant for generalization to older adults. Low-frequency EEG power can be influenced by developmental maturation, sleep and arousal state, medication exposure, medical comorbidity, and aging-related neural changes. Therefore, the present posterior-occipital and broader posterior delta pattern should not be assumed to hold across midlife or older adult ADHD populations without direct replication.

Third, ADHD symptoms and the internalizing covariates were assessed by self-report rather than by clinical interview or multi-informant assessment, which remains the standard basis for clinical diagnosis (American Psychiatric Association, 2013; Kooij et al., 2019). This common-method structure may inflate associations among questionnaire-derived variables and may affect interpretation of residualized ADHD symptom variance. At the same time, the EEG predictor was an independently measured physiological signal rather than another self-report scale.

Fourth, the effect size was small and cross-validated predictive improvement was limited, which is consistent with the interpretation of the finding as a dimensional correlate rather than a prediction model. Fifth, the risk-threshold analysis did not support classification, and prior reviews caution against treating resting EEG summaries as stand-alone diagnostic tools for ADHD (Adamou et al., 2020; Lenartowicz and Loo, 2014; Loo and Makeig, 2012; Boxum et al., 2025). These EEG measures currently lack the sensitivity and specificity required for individual-level screening, diagnostic support, or risk classification.

Sixth, posterior-occipital, posterior, and global delta measures were highly correlated, limiting claims of regional specificity. Future studies should combine resting EEG with task-based measures of sustained attention, inhibitory control, working memory, and arousal regulation; include clinically characterized adult ADHD samples; compare eyes-closed, eyes-open, and task-based states; and test whether the present low-frequency oscillatory pattern predicts longitudinal ADHD-related functioning.

## 6 Conclusion

Higher adult ADHD trait symptoms were associated with lower resting posterior-occipital and broader posterior low-frequency EEG power after adjustment for STAI-T trait anxiety, SDS depressive symptoms, and BAI total score. The association was modest in size but was supported by the planned EEG-family analysis, extended covariate models, robustness checks, and specification-curve analysis. These findings identify a dimensional resting EEG correlate of adult ADHD-related traits. They should be interpreted as evidence for a reproducible group-level association rather than as a biomarker, classifier, screening tool, diagnostic aid, or causal mechanism.

## Data Availability

The raw data supporting the conclusions of this article will be made available by the authors, without undue reservation.
