# Supplementary material for: Resting low-frequency EEG correlates of adult ADHD trait symptoms beyond internalizing symptoms in young adults
Source: Front Hum Neurosci. 2026 Jul 29;20:1878112. doi: 10.3389/fnhum.2026.1878112 (PMC13461718; doi:10.3389/fnhum.2026.1878112)
Supplement: Supplementary file 1 [file Data_Sheet_1.pdf]

Supplementary Material

Resting low-frequency EEG correlates of adult ADHD trait symptoms beyond internalizing symptoms in young adults

This supplement reports ADHD subscale analyses, broad EEG specificity models, robustness checks, spatial collinearity analyses, outcome-specificity checks, risk-threshold analyses, and cross-validation summaries. Unless otherwise noted, covariate-adjusted models use the same deduplicated complete-case framework as the main analyses and HC3 robust standard errors. Throughout this supplement, posterior-occipital delta power refers to the natural-log-transformed mean delta power across PO3, POz, PO4, O1, Oz, and O2, corresponding to the primary EEG marker used in the main analyses.

Table S1. ADHD subscale analyses using posterior-occipital delta power.

Outcome	N	Beta	95% CI	p	q (FDR)
ADHD total	334	-0.114	[-0.199, -0.029]	.008	
Inattention/memory	334	-0.059	[-0.157, 0.038]	.232	.232
Hyperactivity/restlessness	334	-0.112	[-0.210, -0.014]	.025	.065
Impulsivity/emotional lability	334	-0.094	[-0.193, 0.005]	.064	.079
Self-concept problems	334	-0.097	[-0.189, -0.005]	.039	.065
ADHD index items	334	-0.104	[-0.201, -0.006]	.037	.065

Note. All models adjusted for age, sex, STAI-T trait anxiety, SDS depressive symptoms, and BAI total score. Subscale analyses are supportive and should not be interpreted as primary endpoints.

Table S2. Broad EEG specificity models across frequency bands and regions.

Predictor	N	Beta	95% CI	p	q (FDR)
Posterior-occipital delta	334	-0.114	[-0.199, -0.029]	.008	.088
Posterior delta	334	-0.101	[-0.193, -0.008]	.034	.177
Global delta	334	-0.103	[-0.195, -0.011]	.029	.177
Frontocentral delta	334	-0.089	[-0.179, 0.001]	.053	.224
Posterior-occipital theta	334	-0.070	[-0.159, 0.020]	.126	.331
Posterior theta	334	-0.069	[-0.161, 0.023]	.143	.334
Global theta	334	-0.077	[-0.166, 0.013]	.092	.276
Frontocentral theta	334	-0.079	[-0.167, 0.009]	.079	.276
Posterior-occipital	334	-0.034	[-0.117, 0.050]	.426	.895

alpha					
Posterior alpha	334	-0.029	[-0.115, 0.058]	.514	.956
Global alpha	334	-0.017	[-0.103, 0.070]	.708	.956
Frontocentral alpha	334	-0.002	[-0.090, 0.086]	.963	.997
Posterior-occipital beta	334	-0.010	[-0.102, 0.082]	.835	.974
Posterior beta	334	-0.003	[-0.098, 0.092]	.954	.997
Global beta	334	-0.000	[-0.086, 0.086]	.997	.997
Frontocentral beta	334	0.024	[-0.056, 0.103]	.560	.956
Posterior-occipital gamma	334	-0.016	[-0.101, 0.069]	.717	.956
Posterior gamma	334	-0.012	[-0.098, 0.073]	.774	.956
Global gamma	334	-0.023	[-0.109, 0.063]	.597	.956
Frontocentral gamma	334	-0.015	[-0.105, 0.075]	.746	.956
Frontocentral theta/beta	334	-0.101	[-0.173, -0.028]	.007	.088

Note. The broad specificity family was exploratory and included multiple regions and frequency bands. All models adjusted for age, sex, STAI-T trait anxiety, SDS depressive symptoms, and BAI total score.

Table S3. Robustness analyses for the primary association.

Analysis	N	Beta / r	95% CI	p
Primary HC3 model	334	-0.114	[-0.199, -0.029]	.008
Cook's distance <= 4/n (excluded 18)	316	-0.128	[-0.195, -0.060]	< .001
Winsorized 1%/99% outcome and predictor	334	-0.119	[-0.200, -0.038]	.004
Partial Spearman residual correlation	334	-0.125		.022
Permutation partial residual correlation (5000 permutations)	334	-0.154		.003
Bootstrap percentile (2000 resamples)	334	-0.118	[-0.194, -0.037]	.004
Sex interaction: delta × sex	334	0.056	[-0.135, 0.247]	.565
Quadratic term for posterior-occipital delta	334	-0.047	[-0.099, 0.004]	.070

Note. The permutation test and Spearman analyses use partial residual associations. Bootstrap confidence intervals are percentile intervals.

Table S4. Spatial specificity and collinearity checks.

Model	Predictor	N	Beta	95% CI	p
Posterior-occipital delta primary	log_delta_posterior_occipital	334	-0.114	[-0.199, -0.029]	.008

Posterior-occipital delta + global delta	log_delta_posterior_occipital	334	-0.120	[-0.327, 0.088]	.257
Posterior-occipital delta + posterior delta	log_delta_posterior_occipital	334	-0.371	[-0.805, 0.062]	.093
Posterior-occipital delta + frontal delta	log_delta_posterior_occipital	334	-0.074	[-0.197, 0.049]	.240
Posterior delta primary	log_delta_posterior	334	-0.101	[-0.193, -0.008]	.034
Global delta primary	log_delta_global	334	-0.103	[-0.195, -0.011]	.029
Frontal delta primary	log_delta_frontal	334	-0.107	[-0.196, -0.019]	.018
Central delta primary	log_delta_central	334	-0.084	[-0.175, 0.006]	.068

Note. These analyses evaluate whether posterior-occipital delta can be separated from highly correlated posterior/global delta measures. They contextualize topographic interpretation rather than replace the planned primary model.

Table S5. Outcome-specificity checks for posterior-occipital delta power.

Outcome	N	Beta	95% CI	p	q (FDR)
TAI	337	0.094	[-0.020, 0.207]	.106	.484
SDS	336	0.018	[-0.115, 0.152]	.788	.855
BAI	336	-0.007	[-0.168, 0.155]	.933	.933
MPATS	335	0.020	[-0.093, 0.132]	.732	.855
IAT	335	-0.044	[-0.139, 0.052]	.371	.855
Raven	317	-0.014	[-0.120, 0.091]	.790	.855
GSES	334	0.080	[-0.019, 0.179]	.112	.484
PANAS_positive	335	0.095	[-0.017, 0.207]	.095	.484
neuroticism	334	-0.032	[-0.110, 0.047]	.432	.855
extraversion	334	0.042	[-0.084, 0.168]	.512	.855
openness	334	0.033	[-0.077, 0.144]	.554	.855
agreeableness	334	0.018	[-0.081, 0.118]	.720	.855
conscientiousness	334	0.060	[-0.045, 0.166]	.261	.850

Note. Each outcome was modeled with posterior-occipital delta power while adjusting for age and sex. These analyses test whether posterior-occipital delta broadly predicts major psychological covariates.

Table S6. Risk-threshold and cross-validation summaries.

Analysis	N	ADHD / OR	Mean log posterior-occipital delta / CV result
ADHD < 39	302	19.97	0.538
ADHD >= 39	32	43.38	0.527
Logistic model OR	334	OR = 0.761	p = 0.220
Cross-validated delta R2	334	0.0077	95% CI [-0.044, 0.038]

Note. Risk-threshold analyses are descriptive. The logistic model does not support diagnostic classification, individual-level screening, or risk classification.

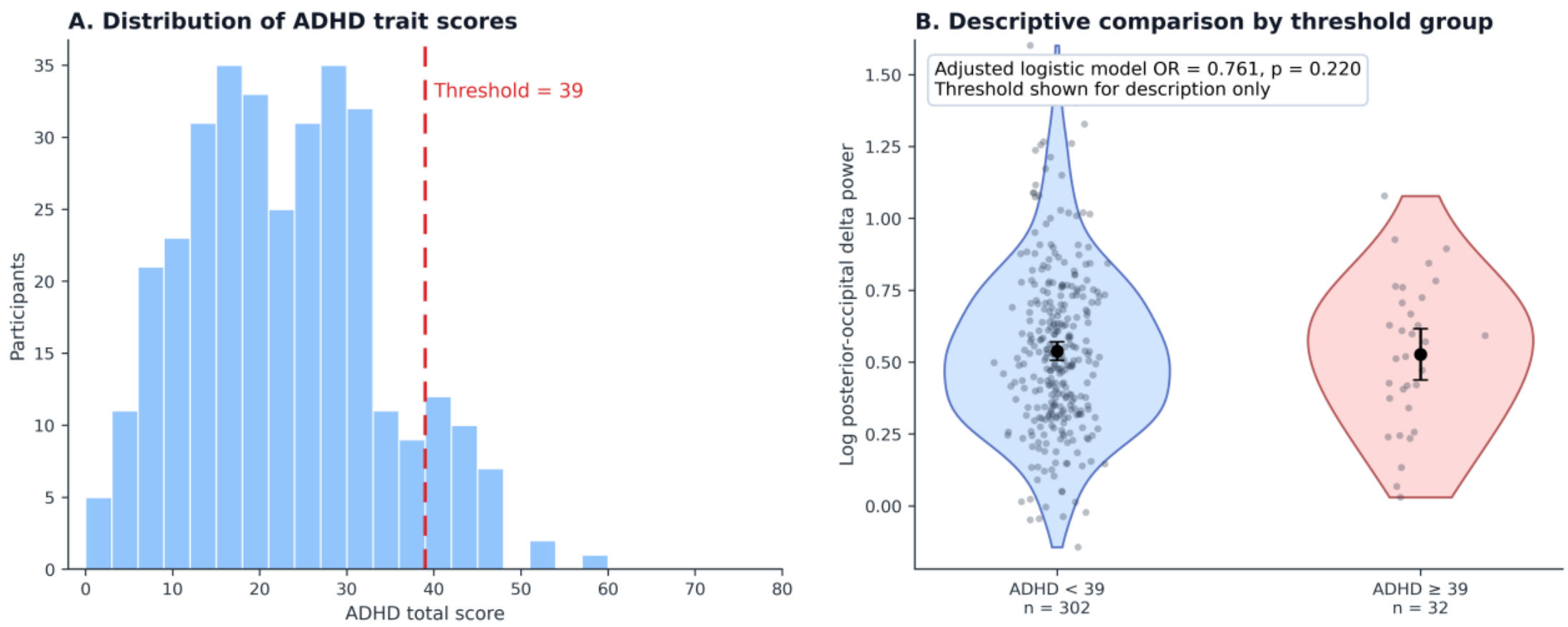

Figure S1. ADHD symptom distribution and descriptive risk-threshold comparison. Panel A shows the distribution of ADHD total scores with the descriptive threshold at 39. Panel B shows the descriptive comparison of log posterior-occipital delta power between participants with ADHD total scores below 39 and those with scores of 39 or higher. Individual observations, group distributions, and mean estimates with 95% confidence intervals are shown. The inset reports the descriptive risk-threshold logistic model. The threshold is included for description only and is not a diagnostic classification, individual-level screening tool, or risk-classification model.

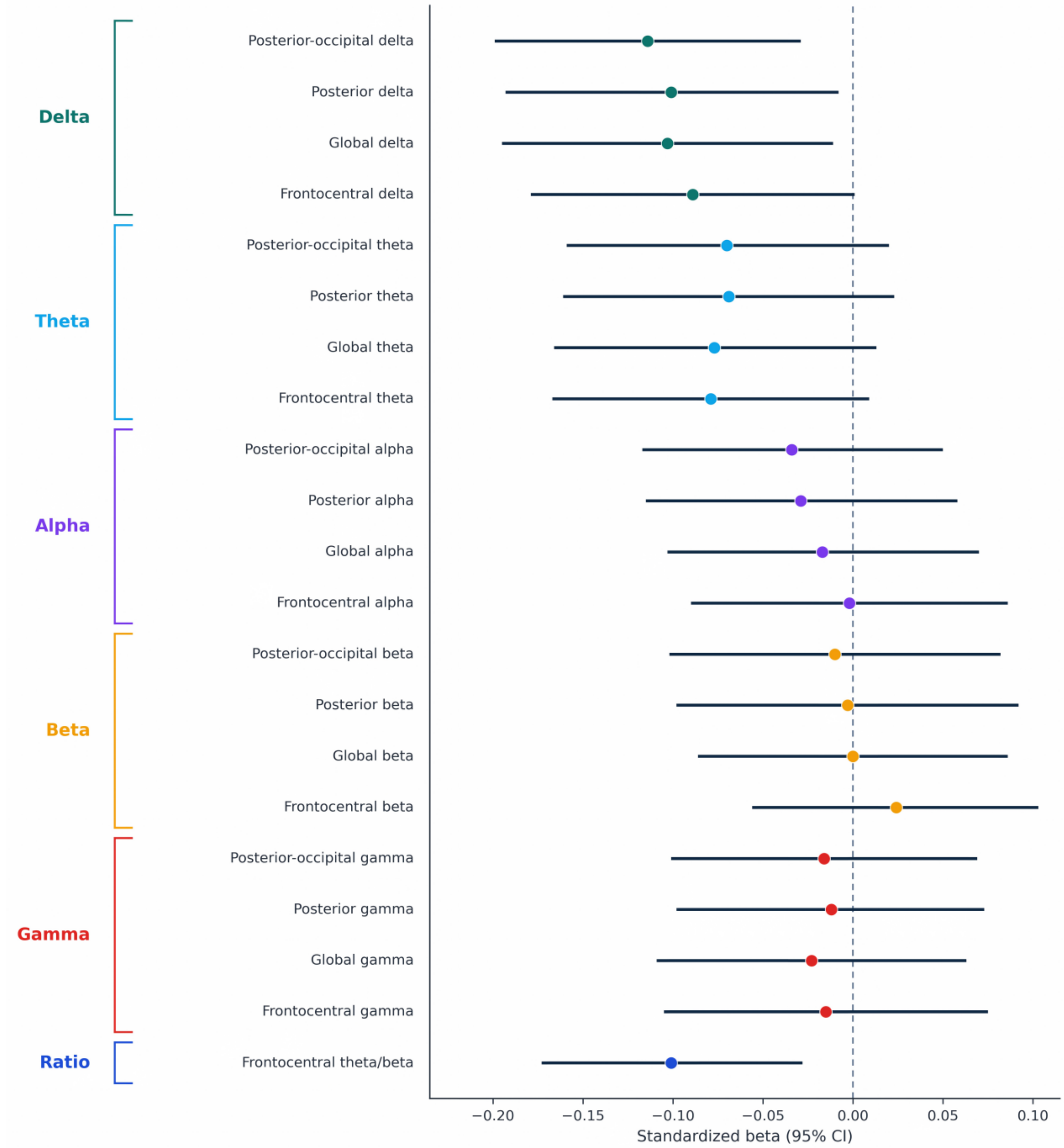

Figure S2. Broad exploratory EEG specificity family. Points are standardized beta coefficients from covariate-adjusted models predicting ADHD total score; bars are 95% confidence intervals. Models adjusted for age, sex, STAI-T trait anxiety, SDS depressive symptoms, and BAI total score. The dashed vertical line marks zero. This exploratory family examined frequency- and region-specific EEG patterns beyond the prespecified four-predictor low-frequency EEG family.

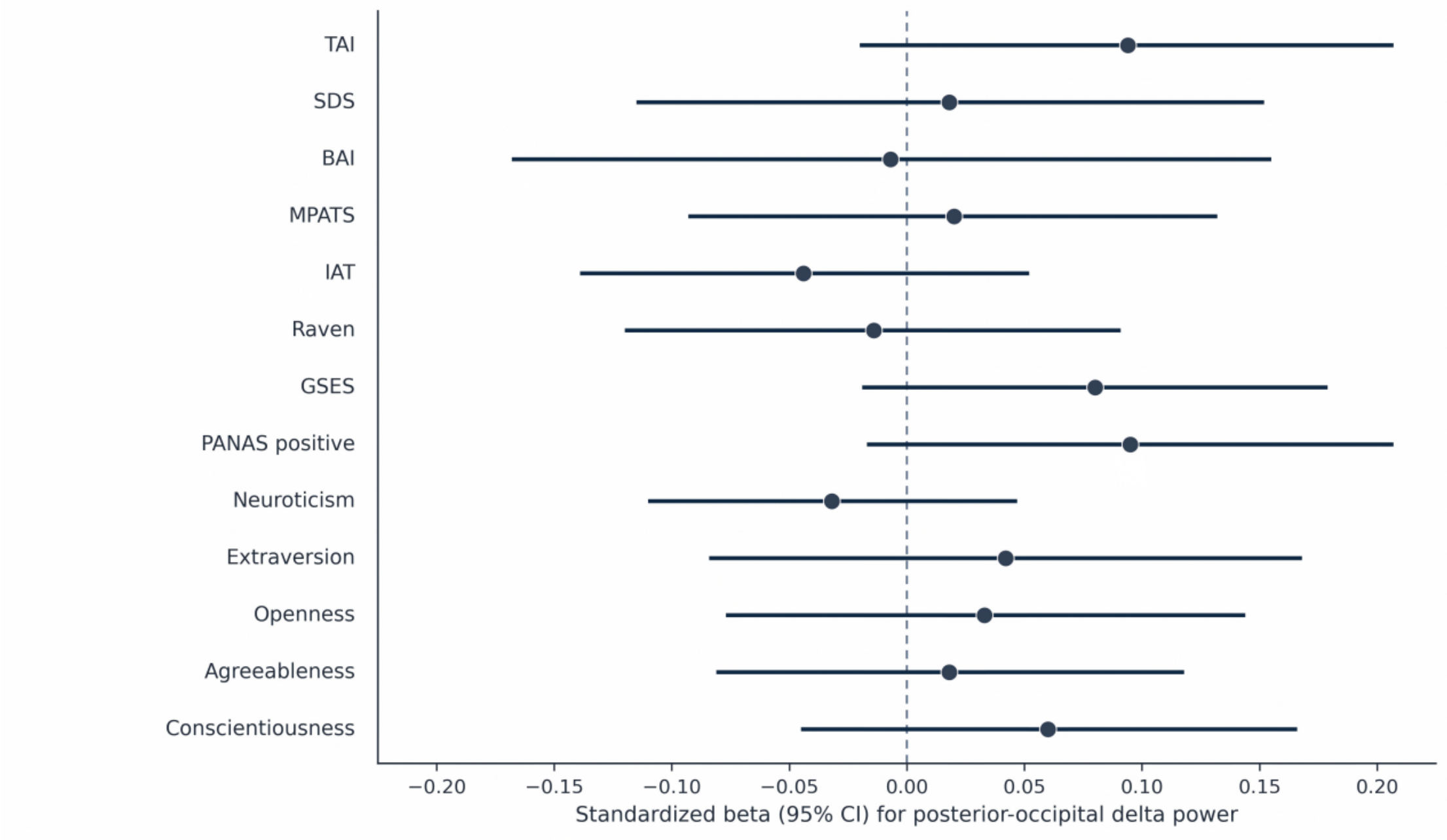

Figure S3. Outcome-specificity checks. Points are standardized beta coefficients for posterior-occipital delta power from age- and sex-adjusted models predicting each psychological or cognitive outcome; bars are 95% confidence intervals. The dashed vertical line marks zero. No association survived FDR correction.
